# Supplementary material for: Are carbon emissions trading and green financial instruments synergistic? -Comprehensive quantitative research based on content analysis
Source: PLoS One. 2024 Mar 7;19(3):e0298601. doi: 10.1371/journal.pone.0298601 (PMC10919601; doi:10.1371/journal.pone.0298601)
Supplement: S1 Data — (ZIP) [file pone.0298601.s001.zip › Data/地级市Z_F工作报告.zip!/╣π╬≈/▒▒║ú╩╨/└┤╫╘╣1⁄2╓┌║┼í╛┬φ┐╦╩2╛▌═°í┐_2015.docx]

各位代表：

现在，我代表北海市人民政府，向大会作政府工作报告，请予审议，并请各位政协委员和其他列席同志提出意见。

一、2014年工作回顾

过去一年，面对严峻复杂的宏观经济形势和历史罕见的台风灾害，全市人民在自治区党委、政府和市委的正确领导下，勇于迎接挑战，奋力开拓进取，经济增长继续领跑全区，经济总量跃居广西沿海三市首位，北海出口加工区B区封关运行，神华国华广投能源基地、诚德冷轧、朗科二期、惠科电子等重大项目开工、投产，教育卫生和保障性住房等民生项目建设力度加大，航空客运突破百万人次，高铁开通运营，银滩大道竣工通车，园博园如期开园，抗击超强台风取得全面胜利，继续保持了北海又好又快的发展局面。

——经济继续保持快速增长。地区生产总值达到856亿元、增长12.5%，是全区14个设区市中唯一完成预期目标并实现两位数增长的城市。财政收入达到127.4亿元、增长12.1%。固定资产投资达到786.2亿元、增长16.5%。规模以上工业总产值达到1597.9亿元、增长22.7%，增加值388.4亿元、增长21.5%。经济发展持续呈现出“速度快、质量好、结构优、后劲足”的鲜明特点。

——三大产业贡献突出。电子信息、石油化工、临港新材料三大产业完成工业产值1312.95亿元、增长19.8%，合计规模以上工业产值占全市总量的80.2%。电子信息产业完成产值810亿元，其中电子信息制造业产值达到721.7亿元，是2008年的15.8倍，为电子信息产业成为广西第10个千亿元产业作出了重大贡献。石油化工产业完成产值345.05亿元，中石化北海炼化公司继续发挥龙头作用，完成产值303.86亿元、税收及地方教育费附加56.1亿元。临港新材料产业完成产值246.2亿元。

——深化改革开放成效明显。政府机构改革、工商登记制度改革等多项改革深入推进。北海出口加工区B区一期工程经过9个月的冲刺，如期建成封关运行。北海机场旅客吞吐量达到100.3万人次，是2008年的3.75倍。北海火车站每天动车开行车次达到13对，旅客到发量超过400万人次，合浦火车站投入使用，玉林至铁山港铁路、铁山港铁路支线完成动态验收，火车首次开进了铁山港。

——生态文明建设深入推进。全面完成节能减排目标任务，万元GDP能耗下降3.2%，万元规模以上工业增加值能耗下降9.3 %。广西最大的园林园艺博览园建成开园，接待游客超过200万人次，获得广泛好评。参加全区“南珠杯”市容环境综合整治竞赛再次获得市、县、镇3个特等奖。开展“美丽北海·生态乡村”活动走在全区前列。

——民生得到有效保障和改善。财政用于民生的支出达77.09亿元，占公共财政预算支出73.44%。筹措24.5亿元实施为民办实事工程，比上年增加10.9亿元。城镇新增就业2.83万人，登记失业率3.11%；农村劳动力转移就业新增1.22万人。城镇居民人均可支配收入达到25818元、增长10.3%，农民人均纯收入达到9079 元、增长10.2%。居民消费价格涨幅控制在2.8%。人民群众分享到了实实在在的发展成果。

一年来，我们坚持“真抓实干、把事干成、造福百姓”的理念，主要做了以下工作：

(一)着力加快产业发展，进一步增强产业支撑

推动新型工业化跨越发展。产业园区共完成产值1483亿元、增长25.9%。坚持以“四定”办法做好项目和投资工作，完成工业投资377.2亿元，三诺智慧产业园、朗科国际存储科技产业园、惠科电子(北海)科技产业园的一期工程全面建成达产，甲骨文公司人才产业基地开业,和润集团项目、神华国华广投北海能源基地等项目开工建设，广西LNG、北海林纸一体化等项目扎实推进，新鑫碳四、和源化工等项目投产，发展后劲显著增强。积极推进创新发展，万人发明专利拥有量增长49.3%。

推动现代服务业繁荣发展。接待国内游客1771万人次、增长16.4%，国内旅游收入增长26.1%，见证了北海30年改革开放历史的富丽华大酒店重新建成营业、成为广西单体规模最大的旅游度假酒店，4A级旅游景区新增2家、总数达到6家，北海老城成为首个文化主题4A级景区，涠洲岛基础设施建设步伐加快，获评为“最美中国旅游目的地城市”。大力发展商贸等服务业，规划建设一批城市综合体项目。着力培育会展经济，成功举办2014北海珍珠展。优化金融环境继续取得明显成效，金融机构贷款余额增长17.8%、增幅位居全区第一，不良贷款率2.33%，不良贷款余额和比率均为2008年以来最低。完成社会消费品零售总额185.8亿元，增长11.2%。

推动特色现代农业加快发展。完成农林牧渔业总产值232.4亿元、增长3.1%，新增农民专业合作社87家、总数达到377家，新增家庭农场41家、总数达到98家。以果蔬、罗汉松、豇豆、夏橙为重点，积极创建现代特色农业示范区。完成水利固定资产投资超过9亿元，解决农村10.1万人的安全饮水问题。拓展远海捕捞作业，赴南沙生产的渔船达152艘。推进新一轮扶贫开发攻坚，贫困人口数量下降至3.82万人，减少了2.01万人。建成通达自然村硬底化道路86条共146公里，完成危桥改造15座。

(二)着力深化各项改革，进一步增强发展动力

认真贯彻落实中央、自治区党委、政府和市委的部署，加快推进重点领域和关键环节的改革。出台和落实扶持措施，促进非公有制经济跨越发展，规模以上非公工业产值增长31.8%，新增产值超百亿元非公企业2家、超50亿元5家。改革工商登记制度，有效激活了社会创业的活力和热情，新设立的市场主体数量增幅名列全区设区市第二位。沿边金融综合改革全面推进，跨境人民币结算量达54.4亿元、增长420.8%，跨境人民币直接投资突破30亿元、占全区比重超过六成。食品药品监管体制改革深入推进，监管能力明显提高。农村土地承包经营权确权试点顺利开展，农村金融改革启动。落实推进北部湾经济区综合改革，户籍、交通、社保等领域的同城化步伐加快。其他改革也取得积极进展。

(三)着力扩大对外开放，进一步增添发展活力

实施更加积极主动的开放带动战略，开放通道建设取得明显成效，合浦至湛江铁路项目广西区内的前置性工作已全部完成，铁山港5－6号公共泊位和中石化北海炼化等3个业主码头建设取得新进展，兰海高速北海段改建工程基本完成，贵港至合浦高速公路、广西滨海公路山口支线开工建设。湛江—北海跨省区特别经济合作区规划启动，龙港新城（香港产业园）概念规划获自治区批复。借鉴上海自贸区“批次进出、集中申报”等监管服务措施，提高了通关效率。外贸进出口总额超过35亿美元、增长30%，其中加工贸易进出口14.2亿美元、增长25.4%。招商引资实际到位资金333.9亿元、增长11.56%，实际利用外资(全口径)3.8亿美元、增长47.3%。

(四)着力完善城市功能，加快建设宜居城市

按照打造生态宜居文明城市的要求，全面加强城市规划、建设和管理工作。基本完成《城市总体规划》修编和一批重要规划的编制工作，完成火车站广场改造和综合整治，积极推进重庆路、广东南路铁路立交、海角路改造等人民群众关注的路网项目。开展主城区内涝整治攻坚行动，完成铁路北侧明渠东段、三江排洪渠等21项应急排水整治工程，解决了一批重要路段的内涝问题。加快推进侨港区域、红湾村等城中村改造，完成北海大道风貌整治工程。努力提升老城区人居环境，完成131条小街小巷改造建设。加快污水垃圾处理设施建设，建成红坎污水处理厂二期工程、涠洲岛污水处理厂、铁山港污水处理厂、云南路生活垃圾转运站等项目。加快发展城市公共交通，开通和优化调整11条公交线路，新增60辆LNG空调公交车，撤销南北二级公路收费站。出租车完成更新换代，服务水平得到提高。全面开展“四车”治理，市区道路交通秩序明显好转。加快创建国家环保模范城市和国家海洋生态文明示范区，启动实施“美丽北海·生态乡村”活动，在全区率先开展铁路沿线环境综合整治并取得显著成效。

(五)着力加强社会建设，做好保障和改善民生工作

坚持优先发展教育，实施教育振兴行动计划，新建、迁建了10所中小学和幼儿园，城区小学一年级平均班额降至54人以下，初中一年级控制在50人以下。义务教育巩固率提高到94%，学前教育三年毛入学率、高中阶段毛入学率分别达80%和91%，高考一本上线率为近年来最高。加强义务教育保障机制建设，接收农民工随迁子女超过1.4万人。全面落实助学政策，资助学生超过10万人次、金额近8000万元。北海艺术设计学院升本成功，北海二本院校增至2所。文化建设取得新成效，海上丝绸之路申遗工作稳步推进，《大浪古城遗址保护规划》编制立项获得国家文物局批准，新建成村级公共服务中心23个。医疗卫生服务水平稳步提高，登革热等疫情联防联控工作取得显著成效，市远程医学中心正式投入运行，银海区人民医院工程完工，总投资4.9亿元的市第二人民医院迁建工程已完成投资1.73亿元，职工医保、城镇居民医保参保率达到98.05%，城乡居民大病保险工作启动。新农合参合率达到99.03%，人均筹资标准增加50元，为农民报销医药费4.4亿元。发放城乡低保金82万人次共1.1亿元，人均补助水平高于自治区标准。新开工建设保障性住房和棚户区改造住房8748套，建成3029套，新增发放租赁补贴763户, 3323户住房困难群众乔迁新居，完成农村危房改造3000户。积极为困难群众提供法律援助，办理法律援助案件1212件。加强平安北海建设，完成平安城市二期项目，加强反恐防暴工作，防范应对能力明显提升，刑事案件发案率下降15.8%。加大打击传销力度，捣毁了一批传销窝点。积极消除影响社会稳定因素，有效预防和化解群体性事件。食品药品安全形势稳定向好，安全生产形势平稳好转，基层应急能力进一步提升，各类突发事件得到有效处置。

(六)着力加强政府自身建设，增强公信力和执行力

深入开展党的群众路线教育实践活动，全面落实中央八项规定精神，坚决整治“四风”，全面加强作风建设，解决了一批基层、企业和群众反映强烈的问题，实现了开展活动与加快发展“两不误、双促进”。进一步精简文件会议，制发文件数量比上年减少19.3%，召开的全市性会议次数减少20%。厉行勤俭节约，全市“三公”经费支出下降27.28%。加强法治政府建设，成立市政府法律顾问团，印发实施21个规范性文件。政府改革取得显著成效，完成市级政府机构改革，推进公共资源交易监管体制改革，做好行政审批事项清理和“接、放、管”工作，清理确认行政审批事项600项，比上年精简17%，下放县区实施363项，取消38项。全市44个具有行政审批职能的部门全部进驻市政务服务中心办理业务，办结各类事项14.73万件，群众满意率达99.98%。全面落实建设工程审批流程优化改革，采用新流程审批项目207个，审批效率显著提高。自觉接受市人大的法律监督、工作监督和市政协的民主监督，办理人大代表建议52件、政协提案317件，办结率和总满意率达100%。

一年来，外事侨务、民族宗教、统计、审计、海事、档案、地方志等工作取得新进展，人口计生、体育、广电、新闻出版、妇女儿童、残疾人等事业取得新进步。国防动员和后备力量建设、海防管控和基础设施建设进一步加强，双拥共建工作取得新成绩。

各位代表!

去年北海遭受了有记录以来登陆强度最大的超强台风“威马逊”以及强台风“海鸥”正面袭击，“威马逊”台风造成全城停电、停水和交通瘫痪，给全市人民生产生活造成了极其严重的影响，全体机关干部、人民解放军、公安民警、武警官兵积极响应市委、市政府号召，率先走上街头，为全市迅速恢复交通秩序，发挥了表率和不可替代的作用。许多老同志和北海市民也纷纷加入了恢复城市正常秩序的行列。供电、供水系统干部职工顶着高温夜以继日工作，为迅速恢复正常生产生活发挥了重要作用。大灾面前，全市干部群众立即行动起来，不畏艰难险阻，为夺取抗灾救灾全面胜利发挥了决定性作用。

各位代表!

过去一年，我们取得了令人自豪的发展成就，巩固和延续了北海2009年以来的良好发展态势。6年来，北海地区生产总值平均增长16.8%、财政收入平均增长29.35%，综合实力显著增强。6年来，在区内各市都在加快发展的情况下，北海主要经济指标在全区的排位大幅上升，其中地区生产总值从第10位跃升到第7位，财政收入从第12位跃升到第6位、增加了100亿元，规模以上工业总产值从第13位跃升到第5位、增加值从第13位跃升到第6位。今日北海，改革开放不断深化、城乡面貌焕然一新、社会事业长足发展、人民生活明显改善、生态建设深入推进，城市发展的内生力显著增强，跳出了“潮起即兴、潮落即衰”的历史循环，“可持续”成为北海发展最突出的特征。我们一定要倍加珍惜北海来之不易的大好局面，不折腾，不自满，一张蓝图绘到底，扎扎实实把北海的事情办好，奋力开创北海更加美好的明天!

各位代表！

我们深知，过去一年的成绩来之不易。这是自治区党委、政府和市委正确领导、科学决策的结果，是市人大及其常委会、市政协和社会各界大力支持的结果，是历届班子历任领导打下良好基础的结果，是全市人民同心同德、艰苦奋斗的结果。我谨代表北海市人民政府，向在座的人大代表、政协委员，向全市干部群众、离退休老同志，向驻市人民解放军、公安民警、武警官兵，向各民主党派、工商联、无党派人士，向各人民团体，向驻市中直、区直单位，向所有关心支持北海发展的港澳台同胞和海内外朋友，表示衷心的感谢和崇高的敬意！

在肯定成绩的同时，我们也清醒地认识到，当前经济社会发展及政府工作还面临不少困难和问题，主要是：受市场因素影响，工业生产增速放缓；现代服务业发展相对滞后；农民稳定增收的难度增大；铁山港口岸正式开放尚未得到解决，产业发展仍然受到物流因素的严重制约；征地搬迁等工作难度加大，融资面临新的困难，有的项目建设进展缓慢；城市建设和管理、民生改善等与人民群众的期待还有不少差距，少数干部作风不实、服务意识不强、办事效率不高的问题依然存在。对此，我们一定高度重视，积极采取措施加以解决。

二、2015年工作安排

总体要求：深入贯彻党的十八大、十八届三中、四中全会、中央经济工作会议和习近平总书记系列重要讲话精神，以邓小平理论、“三个代表”重要思想、科学发展观为指导，坚持“真抓实干、把事干成、造福百姓”的理念，在市委的正确领导下，认真贯彻落实自治区党委、政府的决策部署，主动适应经济发展新常态，坚持稳中求进工作总基调，全面实施《广西北部湾经济区发展规划》，加快发展优势产业，全面深化改革开放，促进城乡协调发展，切实保障和改善民生，推进生态文明建设，加快建设法治政府，保持北海良好发展态势，加快在广西率先全面建成小康社会的步伐。

经济社会发展的主要预期目标：地区生产总值增长10.5%，财政收入增长10%，固定资产投资增长16%，规模以上工业增加值增长17%，城镇居民人均可支配收入增长11%，农村居民人均纯收入增长12%，城镇新增就业人数2.1万人，城镇登记失业率控制在4%以下，居民消费价格涨幅控制在3.0%以下，万元GDP能耗下降1.49%，全面完成自治区下达的节能减排控制性任务。

今年的重点工作：

(一)坚定不移做大做强三大千亿元产业，确保北海经济在新常态下持续较快发展

完成电子信息产业产值1000亿元以上，率先实现打造千亿元产业目标。朗科国际存储科技产业园二期开园，建成惠科电子科技园二期工程，支持建兴、德昌、冠捷、三诺、六禾、创新科等企业上项目、扩产能，确保惠晟液晶面板邦定、世纪创新一体机、嘉信高智能充电等一批中小项目上半年投产。建设广西电子产品检测中心、北海电子信息配套产业园一期，搭建招商平台，提升配套能力。努力克服中石化北海炼化公司停产检修改造和成品油价大幅下滑的影响，力争石油化工产业完成产值300亿元以上，重点加快中石化产品质量升级扩能改造、广西LNG项目建设，推动新鑫碳四、和源化工等项目尽快达产，开工建设中航化、泽天石化等配套项目。扎实做好中石化北海千万吨炼化一体化项目前期工作，争取列入国家“十三五”规划。力争临港新材料产业完成产值350亿元以上，重点加快诚德新材料扩能改造等系列项目建设，加快完善产业链，扩大产业规模。抓好全程跟踪协调和主动服务，加大土地收储力度，加强征地搬迁和用地保障工作，确保北海林纸一体化、和润集团北海项目、神华国华广投北海能源基地、贝因美6万吨液体乳制品等项目建设顺利推进，为进一步扩大北海工业总量夯实基础。促进工业化和信息化深度融合，鼓励企业信息化改造。

(二)大力提高农业产业化水平，促进农民持续增收

加快推进农业产业化。力争农林牧渔业总产值增长5%。加快转变农业发展方式，逐步走出一条“产出高效、产品安全、资源节约、环境友好”的现代农业发展道路。稳定粮食播种面积，提高粮食综合生产能力和粮食安全水平，推进“米袋子”“菜篮子”工程建设。大力发展现代特色农业，扎实创建果蔬产业、罗汉松种植、豇豆种植、夏橙种植加工等4个现代特色农业示范区，核心示范区和拓展区、辐射区农户人均纯收入明显高于当地乡镇农户。加快优质高产高糖糖料蔗基地建设。大力发展林业和特色庭院经济、林下经济。加强农产品流通体系建设，积极发展信息农业、订单农业，促进农产品销售。充分挖掘农业内部增收潜力，开发农村二三产业增收空间，拓宽农村外部增收渠道，加大政策助农增收力度。

夯实农业农村发展基础。加快改善农村生产生活环境，推动“物的新农村”和“人的新农村”建设齐头并进。推进农田水利建设三年攻坚计划，开展乾江围海堤、百曲围海堤、南流江合浦城区防洪整治，全面完成小型水库除险加固，恢复改善农田灌溉面积 8万亩。推进自来水村村通达工程，农村自来水普及率提高至75%以上，加快在全区率先实现自来水村村通目标。加强高标准农田建设，提高耕地质量和综合生产能力。统筹农村电力、通信、有线广电网络、互联网、给排水等管网建设，完善便民利民服务网络。加强农村科技培训，大力培育新型农民。

(三)加快发展第三产业，提升三产对北海经济发展的贡献率

加快发展旅游产业。着力打造北部湾国际旅游度假区核心城市，力争接待国内游客1900万人次。加快景区景点开发，完善旅游服务设施，重点抓好涠洲岛鳄鱼山景区创5A和金海湾等景区创4A工作，加强银滩景区建设管理，打造精品旅游景区。稳步推进涠洲岛整体开发，强化规划引导，突出差异化特色，注重生态环境保护，依法整治岛上违法占地、违法建设行为，建设国际休闲度假海岛目的地。加快中信国安生态旅游、邮轮游艇码头、旅游集散中心、冠岭二期等项目建设，支持合浦县创建广西特色旅游名县。加强旅游整体宣传营销，推进旅游市场秩序综合整治。

加快发展现代物流业等第三产业。坚持生产性服务业与生活性服务业并重、现代服务业与传统服务业并举，积极打造现代服务业集聚区，加快服务业与工农业协同发展、创新发展。紧紧围绕企业生产经营和优势产业发展，大力扶持发展生产性服务业，优化企业发展环境，提升产业竞争力。继续培育定期物流航线，开通北海至越南、马来西亚的国际班轮航线，帮助企业降低物流成本。推动房地产平稳健康发展，鼓励改善性自住等合理需求，促进住房消费。落实国务院促进信息消费扩大内需的战略部署，推进国家信息消费试点城市建设。加快发展金融、商贸、保险和文化娱乐、健康养老、家政服务等服务业，努力改造提升传统服务业。

(四)积极培育新业态和增长点，推动产业优化升级

加快发展海洋产业。认真落实市委关于加快发展海洋经济、建设海洋强市的部署，编制实施北海市海洋经济发展规划，依法保护并合理开发海洋资源，在加快发展临海工业、海洋运输和滨海旅游的同时，促进海洋产业全面繁荣，形成新的增长点。发展船舶修造业，引进大企业建设远洋远海捕捞船队。推动传统渔业向集约型、科技型和深海作业转变，发展高附加值海水养殖业和海产品精深加工业。完善北海内港、南氵万渔港设施，加快营盘渔港建设，打造现代渔港经济区。加快国家级出口养殖水产品质量安全示范区、国家珍珠产业综合标准化示范区建设。推进北海国家农业(海洋)科技园暨北海海洋产业科技园区规划建设，积极引进国家级和高水平的海洋科研机构，打造南方海洋科技城，服务海洋经济发展。

加快发展高新技术产业和各类新型经济业态。大力实施创新驱动战略，推进校企、院企合作，促进科技成果转化应用和产学研融合发展。加快以企业为主体的技术进步和创新体系建设，新增自治区级企业技术中心2家以上，高新技术企业总量达到27家。加快北海软件信息产业园建设，推动甲骨文人才产业基地发展，抓好银河产业城等项目建设，支持石基公司拓展第三方支付业务，壮大软件和信息服务业。积极发展动漫等文化创意产业，大力发展电子商务等互联网经济，支持发展总部经济，逐步形成新型经济业态。

加快发展非公有制经济。坚持大中小并举、扩量与提质并重，落实扶持措施，改进协调服务，优化发展环境，支持有市场有销路的企业扩销促产，帮助有困难的企业走出困境，推动一批大型骨干企业扩能增效、一批中小企业持续壮大、一批小微企业茁壮成长，推动非公经济向规模化、特色化、专业化、高新化发展。

(五)加强城市建设与管理，努力建设人民满意的城市

加强城市规划建设。强化规划引导功能，完成城市总体规划修编和综合交通、市政等7个专项规划编制，积极推进村镇专项规划，加快修编土地利用总体规划，规范城镇集体用地使用管理。编制新型城镇化实施方案，指导推进全市新型城镇化工作。加快北海工业园区产城融合发展和铁山港（临海）工业区区城共建步伐，逐步完善医疗卫生、教育文化、商贸物流等配套设施，推进合浦工业园产城互动试点工作。合理布局和建设一批教育、文化、卫生、体育公共设施，完善提升城市功能，在铁路以南规划建设新的商业圈，加快银滩东区建设步伐，吸引铁路以北城区人口向南转移，促进主城区合理协调发展。加快主城区路网建设，完善“七纵七横”主干路网，建成广东路铁路立交，打通重庆路(广东路至湖南路段)，完成深圳路、湖海路等12条人行道改造，建设上海路铁路立交、西藏路铁路平交、金海岸大道(四川路至北海大道西延线)改造、新世纪大道(武汉路至南珠广场段)、长沙路(新世纪大道至浙江路)。继续抓好城市内涝整治，推进市区内涝整治二期工程并启动三期工程，开工建设铁路北侧排水明渠西段、北海大道西延线排水工程，进一步提升应对内涝能力。加快棚户区、城中村和侨港片区改造，完成一批小街小巷改造工程。实施大县城战略，加快合浦县城开发建设，推动合浦县城和北海主城区一体化发展。促进小城镇加快发展，加快城市公共服务向集镇和农村延伸，分步实施农村规划。加强与北部湾经济区兄弟城市以及湛江、海口等周边城市的互动合作，共同推动环北部湾城市群建设。

加强城市综合治理。继续理顺管理体制，稳步下放管理事权，加快构建“大城管”街面执法格局。建立完善长效机制，持续开展集中整治工作，严厉打击城乡违法用地用海和违法建设、抢建抢种行为。坚持疏堵结合、人性化管理，依法整治占道经营行为。大力完善环卫设施，加强日常管护，建设干净整洁、文明有序的城市。加强交通综合整治，大力发展公共交通，继续严厉打击“四车”等非法营运行为，深入整治出租车拒载、“不打表”现象，不断提升群众满意度。放宽城市建设投资准入，鼓励、支持和引导社会资本采取多种模式平等参与城市公用设施领域的开发、建设和运营。推进扩权强县和扩权强镇，提高县城和小城镇发展能力。逐步将农业转移人口纳入城镇保障性住房体系，提高社会保障、医疗卫生服务水平，保障农民工随迁子女平等接受教育，有序推进农业转移人口市民化。

(六)加快教育文化卫生建设，全面繁荣社会事业

推动教育全面振兴。全面实施北海教育振兴行动计划，筹措资金11亿元推进义务教育均衡发展等八大重点工程，着力改善学校办学条件，促进教育公平，提高教育质量，继续引进优质教育资源，力争学前三年入园率达到81%、九年义务教育巩固率达到 95%、高中毛入学率达到92%，均高于全区平均水平。建成北师大北海附中，力争建成北海十中、海城区十九小、银海区六小和4所幼儿园，启动建设北海十一中。促进高等教育规模化特色化发展。加快发展现代职业教育，扩大优质职业教育办学规模。

加快发展文化体育事业。推动文化事业全面繁荣、文化产业快速发展，增强文化软实力和支撑力。促进基本公共文化服务标准化、均等化，发展文化艺术、新闻出版、广播电影电视、档案、地方志等事业。大力发展群众文化，深入开展文化惠民活动，建设52个村级公共服务中心，组织送戏下乡演出120场次，补贴放映农村公益电影4116场。充分利用园博园设施，积极开展文化活动。扶持文化产业龙头企业，加快近代中西文化系列陈列馆等项目建设。加强文化市场监管，加大扫黄打非工作力度。大力开展全民健身运动，积极发展竞技体育和体育产业，安装健身路径25条，加快推进市体育馆建设前期工作。

加快发展医疗卫生等事业。统筹提高城乡医疗卫生服务能力和水平，继续缓解群众“看病难”“看病贵”问题。开工建设市人民医院异地扩建工程一期、广西海难紧急医学救援中心、市儿童医院、全市急救体系、铁山港区妇幼保健院、合浦县人民医院住院综合楼、合浦县中医院搬迁等项目，完成市第二人民医院迁建主体工程，积极引进优质医疗资源。落实计划生育基本国策，稳定适度低生育水平，促进人口长期均衡发展。完善流动人口管理服务，全面推行居住证制度。加强食品药品监管能力建设，积极创建“食品安全城市”和“农产品质量安全县”，确保人民群众饮食和用药安全。加快其他社会事业发展。

提高社会治理法治化水平。大力弘扬社会主义法治精神，建设社会主义法治文化，形成守法光荣、违法可耻的浓厚社会氛围。完成“六五”普法任务，加快建设覆盖城乡的公共法律服务体系、矛盾纠纷排查化解体系和法律援助服务体系。引导群众依法维权，解决好群众合理合法的利益诉求，形成办事依法、遇事找法、解决问题用法、化解矛盾靠法的良好环境。推进多层次多领域依法治理，发挥社会组织、社会规范等在社会治理中的积极作用。推进平安北海建设，实施网格化管理，创新和健全打防管控长效机制，依法严厉打击暴力恐怖、“两抢一盗”、传销、黄赌毒等违法犯罪活动，持续提升人民群众安全感。全面落实安全生产“党政同责、一岗双责”，抓好烟花爆竹等行业和领域安全隐患的排查整治，坚决遏制重特大安全事故发生。继续加强应急管理规范化建设，提高各类突发事件预防、监控和处置能力。

(七)切实保障和改善民生，让发展成果更多惠及北海人民

着力促进就业创业。多渠道促进充分就业，引导高校毕业生面向基层就业，抓好城镇就业困难人员、农村转移劳动力就业，积极解决被征地农民的就业等生活问题。加强企业用工服务，主动帮助企业解决用工困难。落实创业优惠政策，优化创业环境，促进创业增收致富。主动顺应农民工对身份认同、权益保障、社会融合、价值实现等方面的诉求，帮助农民工更好地就业、创业和发展，新增农村劳动力转移就业0.9万人。

切实做好群众最低生活保障和扶贫开发等工作。扩大城乡社会保障覆盖面，提高城乡低保补助水平，完善基本养老、基本医疗、最低生活保障等社会保障体系。加强社会救助工作，加快发展社会福利和慈善事业，落实优抚双拥安置政策。加强保障性住房建设和管理，逐步满足困难家庭基本需求。继续实施整村推进扶贫开发，创新产业、行业扶贫模式，做到科学精准有效扶贫，全面完成“十二五”扶贫开发任务。

坚持为民办实事。今年要切实办好以下10件实事：1.扩大城市低保保障范围、保障标准从月人均350元提高到420元，月人均补助提高到260元以上；2.扩大农村低保保障范围、保障标准从年人均2000元提高到2600元，月人均补助提高到110元以上；3.新农合筹资标准从每人每年390元提高到450元，其中政府补助从320元提高到360元；4.城镇居民基本医疗保险政府年人均补助从320元提高到360元；5.开展农村居民大病保险补偿，为15000对符合政策的夫妇进行免费孕前优生健康检查；6.资助1.6万名农村义务教育阶段家庭经济困难寄宿生生活费，为1万名中等职业学校在校生免除学费；7.新开工建设公共租赁住房1023套，新增发放租赁补贴180户，改造棚户区(危旧房) 4442户；8.解决农村12万人的安全饮水问题，建设自然村硬化道路100公里，完成800个村屯绿化任务；9.加快银海区果蔬产业示范区建设，建成高标准农业大棚3000亩，辐射带动周边农户种植大棚13000亩，力争核心示范区内农民年人均纯收入达到1.8万元；10.增开8条市区公交线路，新增83辆LNG空调公共汽车，配套建设一批候车亭。

(八)推进全面深化改革，形成推动发展的强大动力

深化政府改革。推进行政审批制度改革，继续取消和下放一批行政审批事项，今后原则上不再新设行政审批事项。完善行政审批事项目录管理，进一步做好“接、放、管”工作，及时全面清理取消非行政许可审批事项，为基层、企业和各种市场主体继续松绑放权。对多个单位共同审批事项进行流程再造，明确一个牵头单位，实行“一个窗口”受理、“一站式”审批、限时办结答复。增强公务人员主动服务意识，推行行政审批“八个办”，让人民群众少跑路就能办成事。完成行政机关和参公管理单位公务用车改革。

深化农村改革。加快农村土地承包经营权、集体建设用地和宅基地确权登记颁证工作。积极发展多种形式农业适度规模经营，培育新型农业经营主体，新增一批专业大户、家庭农场、农民合作社和农业龙头企业，加快构建新型农业经营体系，推进农业经营方式创新。改革农业补贴制度，健全农业支持保护体系。

深化财税金融改革。贯彻落实新的《预算法》，逐步建立全面规范、公开透明的预算制度，让公共财政更好地普惠人民。对中央、自治区和市本级安排专项资金的项目，加强项目进度和支出管理，对长期闲置资金的坚决收回，并启动问责机制。推行政府购买服务，创新公共服务提供机制和方式。加强政府性债务管理，加快建立规范的政府举债融资机制。推进沿边金融综合改革，扩大金融规模，提高市场效率，大力引进金融机构，逐步将北海打造成为北部湾区域性金融中心。积极推进金融机构与企业相互对接，增强金融对项目建设和企业发展的支持力度。深化农村金融改革，大力发展普惠金融，加快建立面向“三农”的多层次、广覆盖、可持续的农村现代金融体系。做好“营改增”和消费税、资源税改革试点工作。

深化社会事业改革。深化教育领域综合改革，着力打造北海教育升级版。深化文化改革，完善文化管理体制和文化生产经营机制，整合用好各类公共文体设施和服务资源，建立健全现代公共文化服务体系。深化医疗卫生体制改革，完善卫生计生机构改革，抓好县级公立医院综合改革，积极推动社会办医，扎实推进全民医保体系建设，完善突发公共卫生事件应急和重特大疾病防控机制。加快推进科技、体育、就业创业、收入分配、社会保障、社会治理、生态文明建设等领域的体制机制改革创新。

积极参与北部湾经济区改革。全面落实自治区党委、政府《关于深化北部湾经济区改革若干问题的决定》，着力抓好户籍、港口、园区、通关、金融及人才开发、行政管理等重点领域和关键环节改革，加快推进北部湾经济区同城化和区域一体化发展，让老百姓和各类市场主体实实在在地享受到更多的便利和好处。

(九)推进全方位开放合作，广泛聚集加快发展的各种要素

开放是北海发展的最大优势，要贯彻落实“一带一路”的战略部署，积极参与共建21世纪海上丝绸之路，编制实施参与共建规划，推进海上互联互通建设、国际产业合作和文化交流。继续做好口岸扩大开放工作，尽快解决制约铁山港发展的瓶颈问题。充分利用北海出口加工区保税物流功能，积极复制上海自贸区监管服务措施，加快电子口岸建设，促进口岸通关便利化，提高通关效率。积极参与“美丽中国--丝绸之路旅游年”活动，支持开展北海—东盟海上丝绸之路邮轮旅游。推进海上丝绸之路联合申遗，加快合浦汉文化公园建设，加强文化遗产发掘、保护和利用，积极开展对外宣传，扩大北海作为国家历史文化名城的影响力。

推进区域合作发展。抓好招商引资工作，积极承接产业转移。创新利用外资方式，扩大利用外资规模。建好和用好国家科技兴贸、加工贸易梯度转移、外贸转型升级等基地平台，深入实施加工贸易三年倍增计划，外贸进出口总额达到40亿美元以上。继续扩大面向粤港澳台的开放合作，推进湛江—北海跨省区特别经济合作区和龙港新城(香港产业园)规划建设，努力在推动两广一体化中发挥更大作用。加强与西南、中南的合作，推进“北海—贵阳港”等项目建设。

加强开放通道和合作平台建设。重点推进合浦至湛江铁路以及铁山港1-4号泊位专用线、铁山港至石头埠支线等铁路项目，增加进出北海的动车车次，争取增开城际列车，推动北海火车站升级为一等客货运站，形成大能力的出海客货铁路通道，加快发展高铁经济。力争建成铁山港5-6号公共泊位、石头埠1-2号泊位和中石化成品油码头、广西LNG码头，开工建设铁山港航道二期扩建工程和7-8号公共泊位。继续培育航空市场，提升北海机场辐射服务能力。加快贵港至合浦高速公路等项目建设，做好滨海公路(大风江至高德段)等项目前期工作。加强北海出口加工区B区招商工作，启动B区二期工程前期工作，开工建设北海报关报检查验中心，力争北海高新区升级为国家级高新区、铁山港(临海)工业区获批为自治区级开发区。

(十)大力开展“美丽北海·生态乡村”活动，全面推进生态文明建设

深入推进“美丽北海”乡村建设活动，在巩固“清洁乡村”成果的基础上，筹措资金2.26亿元，全面实施“美丽北海·生态乡村”建设，突出抓好村屯绿化、饮水净化、道路硬化，持续优化乡村生态环境。推进四川路至合浦火车站铁路沿线环境风貌整治，逐步把铁路沿线建设成为北海亮丽的风景线。完善主城区污水泵站和管网建设，建成南康、营盘、福成等污水处理厂及配套管网，全面完成“十二五”城镇污水垃圾处理设施建设任务。加强海洋资源环境保护，推进涠洲岛珊瑚礁国家级海洋公园建设，扎实创建国家级海洋生态文明示范区。深入开展机动车污染减排，全面落实防尘降尘措施，保持优良的空气质量。实行最严格的水资源管理制度，加强南流江水域、牛尾岭水库和星岛湖环境整治与保护，加大饮用水水源地保护力度，启动城市备用水源建设。大力发展生态经济和循环经济，积极倡导低碳生活。抓好资源能源节约和污染物总量减排工作，全面完成应对气候变化和节能减排任务。

各位代表!

今年是实施“十二五”规划的最后一年，我们将努力完成“十二五”规划的目标任务，切实做好“十三五”规划编制工作，为“十三五”的发展奠定重要基础。

三、深入推进依法行政，加快建设法治政府

依法全面履行政府职能。坚持法定职责必须为、法无授权不可为，全面清理政府部门权力，明确政府部门责任，并按自治区的统一部署，逐步制定公布权力清单、责任清单、负面清单和财政专项资金管理清单。抓好落实是对政府工作的基本要求，政府系统要紧紧围绕市委的决策部署，在抓落实上狠下功夫，全面提高执行力和公信力。全体工作人员要牢固树立大局意识、法治意识、规则意识和责任意识，讲政治、守规矩，干实事、办成事、惠民生。

强化依法决策。认真落实市委“三重一大”事项决策试行办法的要求和市人大常委会讨论决定重大事项的相关规定。全面推行政府法律顾问制度，完善政府内部决策规则，严格履行公众参与、专家论证、风险评估、合法性审查和集体讨论决定的法定程序，把重大行政决策全程纳入法治化轨道。

严格公正文明执法。深化行政执法体制改革，推进简政放权，加强县区政府执行职责，积极改善基层工作条件，提高公共服务能力。加强综合执法机构建设，减少多头执法。建立乡镇国土、住建、环保、安监“四所合一”管理体制，充实县区基层行政执法力量。落实行政执法人员持证上岗和资格管理制度，严格规范执法程序。

加强行政权力制约和监督。依法自觉接受市人大及其常委会的法律监督和工作监督，认真办理人大代表意见建议。自觉接受市政协的民主监督，认真办理政协提案。保障和支持审计机关、监察机关依法开展专门监督，做到有错必纠、有责必问。建立行政执法公示制度，主动向社会公开涉及社会公共利益、公众普遍关注、需要社会知晓的执法信息。深入推进政府信息公开，构建覆盖市县(区)镇(乡、街道办)村(居委会)的四级政务服务体系。

努力打造高效廉洁的公务员队伍。认真贯彻市委十届五次全会和市十届纪委五次全会精神，激发广大干部的干事创业热情，让干部始终把使命铭记心中、把责任扛在肩上。强化政府部门间的沟通、协调与配合，进一步树立服务意识，优化政务环境，提升行政效能。持之以恒落实中央八项规定精神，认真践行“三严三实”，深入整治“四风”，严格遵守“约法三章”、厉行节约、公务接待等规定，坚决纠正不作为、乱作为，坚决克服懒政、怠政，坚决惩处失职、渎职。全面落实党风廉政建设“一岗双责”，深入推进惩治和预防腐败体系建设，坚决查处腐败行为，加强对干部的廉政教育，使干部不敢腐、不能腐、不想腐，树立政府公务人员为民务实清廉的良好形象。

各位代表!

“人民对美好生活的向往就是我们的奋斗目标”，人民对政府工作的期待就是我们的努力方向，人民对政府的信任支持就是我们的前进动力。我们相信，在自治区党委、政府和市委的正确领导下，通过全市人民的共同努力，我们一定能够全面完成今年和“十二五”的各项目标任务，不断谱写北海改革发展的新篇章!
